# Dynamics of clusterin protein expression in the brain and plasma following experimental traumatic brain injury

**DOI:** 10.1038/s41598-019-56683-6

**Published:** 2019-12-27

**Authors:** Shalini Das Gupta, Anssi Lipponen, Kaisa M. A. Paldanius, Noora Puhakka, Asla Pitkänen

**Affiliations:** 0000 0001 0726 2490 grid.9668.1 A. I. Virtanen Institute for Molecular Sciences, University of Eastern Finland, PO Box 1627, FI-70211 Kuopio, Finland

**Keywords:** Neurodegeneration, Trauma

## Abstract

Progress in the preclinical and clinical development of neuroprotective and antiepileptogenic treatments for traumatic brain injury (TBI) necessitates the discovery of prognostic biomarkers for post-injury outcome. Our previous mRNA-seq data revealed a 1.8–2.5 fold increase in clusterin mRNA expression in lesioned brain areas in rats with lateral fluid-percussion injury (FPI)-induced TBI. On this basis, we hypothesized that TBI leads to increases in the brain levels of clusterin protein, and consequently, increased plasma clusterin levels. For evaluation, we induced TBI in adult male Sprague-Dawley rats (n = 80) by lateral FPI. We validated our mRNA-seq findings with RT-qPCR, confirming increased clusterin mRNA levels in the perilesional cortex (FC 3.3, p < 0.01) and ipsilateral thalamus (FC 2.4, p < 0.05) at 3 months post-TBI. Immunohistochemistry revealed a marked increase in extracellular clusterin protein expression in the perilesional cortex and ipsilateral hippocampus (7d to 1 month post-TBI), and ipsilateral thalamus (14d to 12 months post-TBI). In the thalamus, punctate immunoreactivity was most intense around activated microglia and mitochondria. Enzyme-linked immunoassays indicated that an acute 15% reduction, rather than an increase in plasma clusterin levels differentiated animals with TBI from sham-operated controls (AUC 0.851, p < 0.05). Our findings suggest that plasma clusterin is a candidate biomarker for acute TBI diagnosis.

## Introduction

Traumatic brain injury (TBI) affects an estimated 69 million individuals worldwide each year, including approximately 1.7 million in the USA and 2.5 million in Europe^1–3^, and around 40% of TBI patients suffer long-term disabilities^4^. Despite a large number of favourable preclinical proof-of-concept trials, no clinical treatments are yet available to improve post-TBI outcome^5^. One major reason for the stalled translation of data from the laboratory to the clinic is the lack of biomarkers for subject stratification and therapy follow-up.

TBI is a dynamic condition consisting of temporally overlapping molecular and cellular pathologies^6,7^. Impact-related injuries induce changes in the extracellular ion balance and transmitters, haemorrhage, and tissue oedema^8^. Secondary injuries include neurodegeneration, neurogenesis, axonal sprouting, axonal and myelin injury, dendritic remodelling, various types of gliosis, inflammatory cell invasion, blood-brain-barrier damage, angiogenesis, alterations in extracellular matrix composition, possible aggregation of materials (*e.g*., iron and calcium), and acquired channelopathies^9,10^. Biomarker studies have largely focused on the secondary injury mechanisms, which progress over hours to months, eventually in parallel with the activation of “self-repair” pathways^11,12^. For clinical application, the biomarker should be non-invasive^13^. Blood-based biomarkers hold promise as they can elucidate the ongoing TBI-induced molecular alterations in the brain^14^. Further, obtaining blood samples is less invasive than cerebrospinal fluid sampling (CSF) sampling and is routinely performed in clinical settings.

Clusterin is a highly conserved glycoprotein ubiquitously expressed in the brain as well as in peripheral tissues in different species, including rodents and humans^15^. It is also a soluble complement cascade-regulating factor^16^, and plays a major role as a chaperone^17,18^ and in regulating cellular apoptosis^19–21^. Clusterin expression is low in the normal brain^16^, whereas its levels are markedly increased in both experimental models and humans with neurodegenerative or neurodevelopmental disorders, as well as after hypoxic-ischemic brain insult^22,23^. Several recent studies demonstrated that CSF and circulatory clusterin levels serve as a prognostic and/or diagnostic biomarker for neuropathologies such as Alzheimer’s disease (AD), mild cognitive impairment, multiple sclerosis, acute ischemic stroke, and epilepsy^19–31^. Although previous studies of AD suggest that the plasma clusterin level indicates the brain amyloid load, a shared pathology between TBI and AD^16,25,32–35^, few studies have evaluated clusterin expression in the brain and biofluids following TBI.

Our previous mRNA-seq studies revealed increased clusterin mRNA levels in the perilesional cortex and thalamus at 3 months after lateral fluid-percussion injury (FPI)-induced TBI in rats^36^. We also observed elevated clusterin mRNA levels in the perilesional cortex and thalamus of both wild-type C57BL/6J and the APP/PS1 mouse model of AD after severe controlled cortical impact (CCI)-induced brain injury^37^. Based on these initial findings, we hypothesised that an increase in clusterin protein levels could indicate injury in the lateral FPI model of TBI, and that the increased brain levels of clusterin would lead to elevated plasma clusterin levels, making it a potential non-invasive prognostic biomarker for TBI-induced brain injury. Therefore, in the present study, we assessed the spatio-temporal dynamics of clusterin protein expression in the brain at both acute and chronic time-points post-TBI. Moreover, we investigated whether the levels of secreted plasma clusterin distinguish TBI rats from naïve or sham-operated controls.

## Results

### Punctate clusterin immunoreactivity showed region-specific temporo-spatial expression in the perilesional cortex, ipsilateral hippocampus, and thalamus

**Figure 1:**
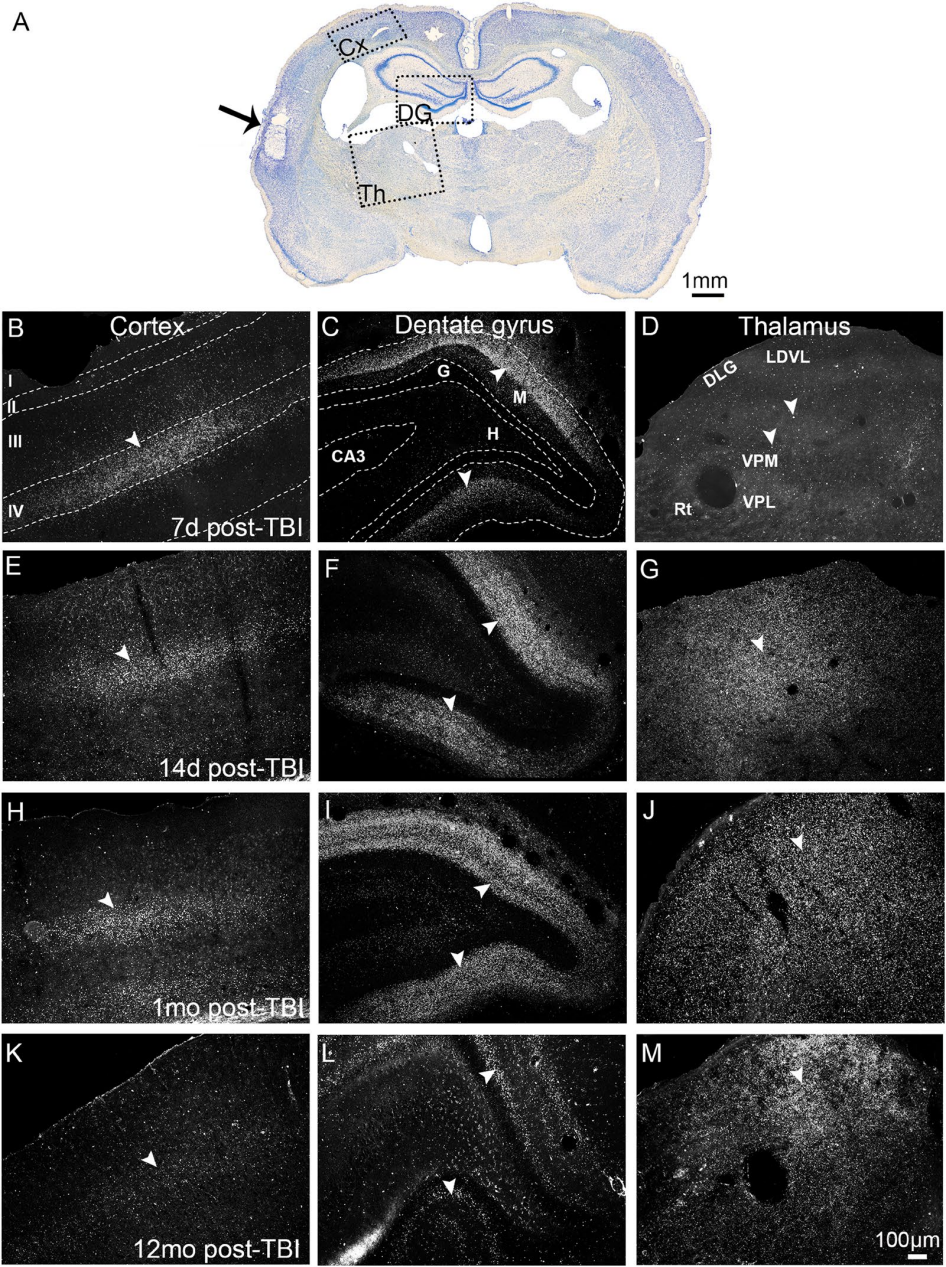
Temporo-spatial evolution of prolonged clusterin immunoreactivity in the cortex, dentate gyrus, and thalamus ipsilateral to the injury. **(A)** A brightfield photomicrograph of a Nissl-stained coronal section from a rat perfused for immunohistochemistry at 1 month after TBI. The lesion core is indicated by an arrow. Dashed boxes indicate the brain regions with prominent clusterin immunoreactivity (ir) in layer IV of the ipsilateral cortex (Cx; panels B,E,H,K), molecular layer of the dentate gyrus (DG; panels C,F,I,L), and dorsal aspect of the thalamus (Th; panels D,G,J,M). Higher-power darkfield photomicrographs show that at 7 d post-TBI, punctate clusterin immunoreactivity (arrowheads) was prominently present in **(B)** layer IV of the perilesional cortex, **(C)** in the molecular layer of ipsilateral dentate gyrus, and faintly in **(D)** the ipsilateral dorsal thalamus. At 14 d post-TBI, **(G)** the thalamic immunostaining increased. Cortical **(E,H)** and hippocampal **(F,I)** staining persisted prominently for up to 1 month post-TBI, becoming substantially weaker by **(**cortex **K**, hippocampus **L)** 12 months post-TBI. In contrast, **(J,M)** thalamic staining remained prominent from 1 month until 12 months post-TBI. No immunostaining was observed in the corresponding contralateral brain areas. Abbreviations: CA3, cornu Ammonis 3; d, days; DLG, dorsal lateral geniculate nucleus; G, granule cell layer of the dentate gyrus; H, hilus; LDVL, laterodorsal thalamic nucleus, ventrolateral part; M, molecular layer of the dentate gyrus; mo, months; Rt, reticular thalamic nucleus; TBI, traumatic brain injury; VPL, ventral posterolateral thalamic nucleus; VPM, ventral posteromedial thalamic nucleus. Scale bar equals 100 µm for all panels.

We analysed the temporospatial expression of clusterin immunoreactivity (ir) in the brain after lateral FPI-induced TBI at several time-points post-TBI. At 2 d post-TBI, no clusterin-ir was detected in the perilesional cortex, hippocampus, or thalamus. From 1 wk to 12 months post-TBI, prolonged spatio-temporally regulated clusterin-ir was observed in the ipsilateral cortex, dentate gyrus, and thalamus (Fig. 1A).

At 1 wk post-TBI, we observed a very high density of punctate immunolabeling in layer IV of the perilesional cortex and in the molecular layer of the ipsilateral dentate gyrus (Fig. 1B,C**)**. In sections sampled at 2 wk to 1 month post-TBI, layer IV of the perilesional cortex and the molecular layer of the ipsilateral dentate gyrus remained intensively immunolabelled (Fig. 1E,F,H,I). In the thalamus, the post-TBI evolution of immunolabeling appeared more slowly than in the perilesional cortex and dentate gyrus. Ipsilateral thalamus was only lightly immunolabelled at 1 wk post-TBI (Fig. 1D), but the intensity of the immunopositive labelling progressively increased from 2 wk to 1 month post-TBI, particularly in the latero-dorsal aspect of the ipsilateral thalamus (Fig. 1G,J).

At 12 months post-TBI, the lateral and dorsal aspects of the ipsilateral thalamus exhibited prominent clusterin-ir (Fig. 1M). The molecular layer of the ipsilateral dentate gyrus was also positively immunostained (Fig. 1L). The perilesional cortex showed comparatively weaker immunoreactivity with light punctate immunolabeling in layer IV (Fig. 1K).

Contralateral punctate clusterin-ir was not observed in the TBI rats at any time-point post-injury. Further, there was no positive clusterin-ir in the cortex, hippocampus, or thalamus in the sham-operated rats at any time-point (Supplementary Fig. S1). In both sham-operated and TBI rats, we observed clusterin-ir bilaterally in the lateral hypothalamus, paraventricular and supraoptic nuclei of the hypothalamus, and median eminence. In contrast to the punctate extracellular clusterin-ir observed in the ipsilateral cortex, hippocampus, and thalamus in the TBI rats, the hypothalamic staining was predominantly in the cell bodies, and cytoplasmic in nature. We also observed clusterin-ir varicose axonal labelling from the paraventricular nucleus toward the supraoptic nucleus and hypophysis (Supplementary Fig. S2).

### Clusterin immunolabeling did not colocalise with neuronal, glial, or mitochondrial markers

**Figure 2:**
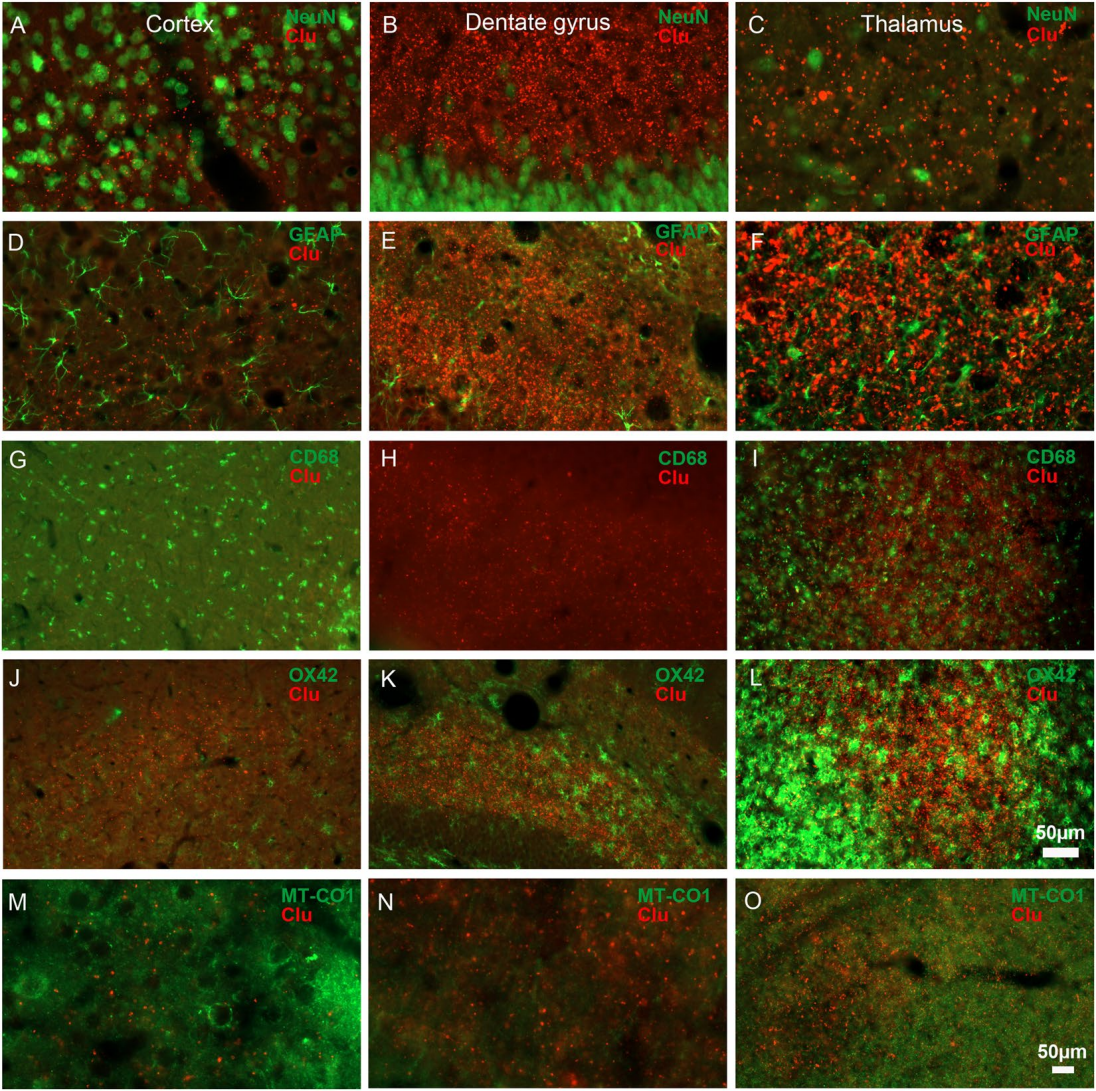
Post-TBI expression of clusterin is prominent in the extracellular space. Double-immunofluorescence revealed that in the perilesional cortex, ipsilateral dentate gyrus, and ipsilateral thalamus, clusterin-immunoreactivity did not colocalise with the **(A–C)** neuronal marker NeuN, **(D–F)** astrocyte marker GFAP, microglial markers **(G–I)** CD68 or **(J–L)** OX42, or **(M–O)** the mitochondrial marker MT-CO1. In the ipsilateral dorsal thalamus, robust clusterin immunoreactivity surrounded the cells labelled with microglial markers **(I)** CD68 and **(L)** OX42, and **(O)** mitochondria labelled with MT-CO1. All images were taken from rats perfused at 1 month post-TBI. Abbreviations: CD68, cluster of differentiation 68; Clu, clusterin; GFAP, glial fibrillary acidic protein; MT-CO1, mitochondrially encoded cytochrome c oxidase 1; NeuN, neuronal nuclei; OX42, antibody against CD11b/c; Scale bar = 50 µm for all panels.

Next, we investigated the cellular and subcellular localisation of clusterin-ir. Because clusterin-ir was most prominent in all the three injured brain areas (perilesional cortex, ipsilateral dentate gyrus, and thalamus) at 1 month post-TBI, we selected this time-point to perform colocalisation analyses. Punctate clusterin-ir in the perilesional cortex, ipsilateral dentate gyrus, and ipsilateral thalamus did not colocalise with the neuronal marker NeuN (Fig. 2A–C), astrocyte marker glial fibrillary acidic protein (GFAP) (Fig. 2D–F), or the microglial markers CD68 (Fig. 2G–I) and OX42 (Fig. 2J–L). In addition, there was no colocalisation with the mitochondrial marker MT-CO1 (Fig. 2M–O).

A high density of diffuse punctate clusterin-ir in the ipsilateral thalamus, however, was observed adjacent to and surrounding the intense labelling of the microglial markers CD68 and OX42 (Fig. 2I,L), and the mitochondrial marker MT-CO1 (Fig. 2O), indicating increased extracellular clusterin expression in the vicinity of ongoing chronic thalamic microglial and mitochondrial activation after TBI.

### Increased clusterin protein expression corresponded with increased *Clu* mRNA levels in the brains of the TBI-induced rats

Our previous RNA-seq analysis of TBI-induced rats at 3 months post-injury revealed a 2.5-fold increased *Clu* mRNA expression in the ipsilateral cortex and 1.8-fold in the thalamus, whereas the hippocampus showed only a trend toward higher *Clu* mRNA levels^36^.

**Figure 3:**
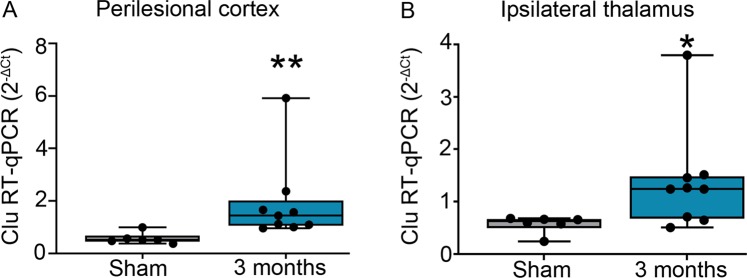
Elevated *Clu* mRNA expression was observed in the brain at 3 months post-TBI. TaqMan RT-qPCR analysis revealed increased *Clu* mRNA expression in the **(A)** perilesional cortex (FC 3.3, p < 0.01) and **(B)** ipsilateral thalamus (FC 2.4, p < 0.05) of the rats at 3 months post-TBI as compared to sham-operated controls (n = 9 TBI, 6 sham; each dot in panels A-B refers to one animal). Clusterin Ct values were normalised to the housekeeping gene GAPDH. Statistical significances: *p < 0.05; **p < 0.01 (Mann-Whitney U test). Abbreviations: Clu, clusterin; Ct, cycle threshold; RT-qPCR, reverse transcription-quantitative polymerase chain reaction; sham, sham-operated controls.

In this study, we validated our previous RNA-seq findings using reverse transcription-quantitative polymerase chain reaction (RT-qPCR). RT-qPCR confirmed the increased Clu mRNA levels in the ipsilateral cortex (FC 3.3, p < 0.01, Fig. 3A) and ipsilateral thalamus (FC 2.4, p < 0.05, Fig. 3B) in TBI rats compared with sham-operated controls.

### Plasma clusterin levels were acutely down-regulated post-TBI, rather than chronically elevated

**Figure 4:**
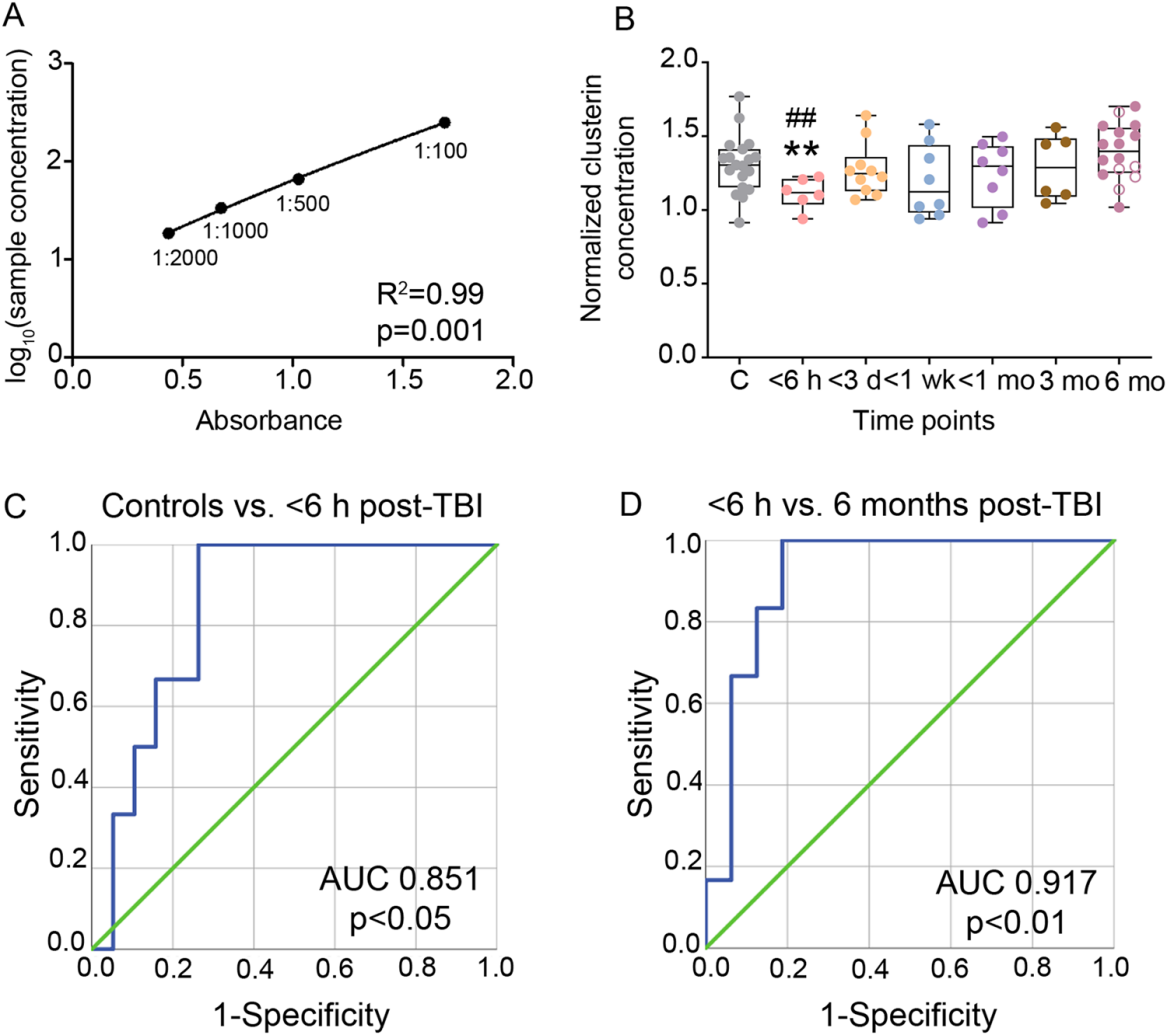
Acute reduction in plasma clusterin levels after TBI. (**A**) The dilution curve demonstrated linearity, indicating no significant matrix interference effect in the ELISA assay. **(B)** Clusterin levels in plasma derived from the cardiac puncture at very acute post-TBI time-points (<6 h, *i.e*., 2 h-6 h post-TBI) were lower than that in controls (15%, p < 0.01) or that at 6 months post-TBI (21%, p < 0.01). At the other time-points, there was no difference between the TBI and control animals, or within the TBI groups. Moreover, at 6 months post-TBI, the plasma clusterin levels did not distinguish the rats with (open circles) or without (filled circles) spontaneous seizures (p > 0.05). **(C)** ROC analysis indicated that plasma clusterin levels sampled <6 h from TBI distinguished the animals with TBI from controls with an AUC of 0.851 (p < 0.05) and **(D)** from the 6 months post-TBI group with an AUC of 0.917 (p < 0.01). Statistical significances: **p < 0.01 compared with the control group; ##p < 0.01 compared to 6 months post-TBI (Mann-Whitney U test). Abbreviations: AUC, area under curve; C, controls; d, days; h, hours; mo, months; R^2^, coefficient of determination; wk, weeks.

To analyse linearity within the sensitivity range of the enzyme-linked immunosorbent assay (ELISA) kit, we prepared a 4-point dilution curve from a test plasma sample. The clusterin concentration exhibited a linear decrease with increasing dilution factor (R^2^ = 0.99, p = 0.001, Fig. 4A), particularly within the dilution range of 1:500–1:2000, indicating that the assay was sensitive in this range for evaluating the clusterin concentration. A 1:500 dilution factor was selected for further analysis.

After confirming that haemolysis in cardiac plasma samples did not affect the clusterin ELISA assay (Supplementary Fig. S3), we investigated whether post-TBI increases in clusterin levels in the multiple brain areas were associated with increases in plasma clusterin levels.

The clusterin levels in cardiac plasma did not differ significantly among the naïve rats and sham-operated experimental controls at any time-point (p > 0.05). Hence, these animals were combined to form the control group.

The TBI animals were grouped as follows: blood sampling between 2–6 h (<6 h), 1–3 d (<3 d), 5 d-1 wk (<1 wk), 2 wk-1 month (<1 mo), 3 months, and 6 months post-TBI. At the most acute time-point post-TBI (2–6 h), plasma clusterin levels were reduced in TBI animals compared with controls (FC 0.85, p < 0.01) and the 6 months post-TBI group (FC 0.79, p < 0.01) (Fig. 4B). Receiver operating characteristic (ROC) analysis revealed an area under the ROC curve (AUC) of 0.851 (p < 0.05, normalised clusterin concentration cut-off of 1.22 resulted in 83% sensitivity and 74% specificity, Fig. 4C) when comparing the <6 h group with the controls. The <6 h group also differed from the 6 months post-TBI group with an AUC of 0.917 (p < 0.01, normalised clusterin concentration cut-off of 1.22 with 83% sensitivity and 88% specificity, Fig. 4D). No significant difference was detected among the other post-TBI time-points, or when these TBI groups were compared with controls.

The 6 months post-TBI group also included epileptic rats. Spontaneous seizures were observed in 31% of the rats (5/16), whereas 69% exhibited no seizures (11/16). The plasma clusterin concentrations did not differ between the epileptic and non-epileptic rats (FC 0.92, p > 0.05).

### Clusterin levels were elevated in the rat CSF at 12 months post-TBI

Because only small volumes (typically 50–120 µl) of CSF can be withdrawn from the cisterna magna of rats, we limited our analysis of CSF clusterin levels to animals that had been followed-up for 12 months post-TBI. As described earlier, the same rats had elevated clusterin-ir in the thalamus, perilesional cortex, and ipsilateral dentate gyrus. Moreover, at this chronic time-point, injury-related intracerebral haemorrhage was no longer a problem for CSF protein analysis.

The CSF proteomics indicated a 1.3-fold increase (TBI/sham ratio, FDR-corrected p value = 0.02) in the clusterin protein level at 12 months post-TBI (Supplementary Table S1).

## Discussion

In the present study, we evaluated whether clusterin expression was increased in the rat brain after lateral FPI–induced TBI, a model of closed-head injury in humans, resulting in increased plasma clusterin levels, which could then be used as a non-invasive diagnostic biomarker for TBI and a prognostic biomarker for the extent of brain injury. We had three major findings. First, expression of clusterin protein was increased from 1 week until 12 months post-TBI, but the timing of peak expression varied in the perilesional cortex, dentate gyrus, and thalamus. Second, clusterin-ir did not colocalise with neuronal or glial markers, but intense clusterin immunolabelling was observed in the extracellular space and select axonal pathways. Third, the increased brain levels were associated with reduced rather than increased plasma levels of clusterin after TBI.

The major finding of the present study was that TBI induces a robust clusterin expression that is long-lasting and temporally regulated in different injured brain areas. Importantly, immunostaining with both monoclonal and polyclonal primary antibodies raised against clusterin revealed comparative punctate immunoreactivity restricted to the brain areas ipsilateral to the injury. There was no positive immunoreactivity in the contralateral brain areas of the rats with TBI and in the sham-operated controls. These data expand the previous observations in rodent models and human TBI. Consistent with our findings, a previous study implementing a weight-drop model of cortical contusion in adult female Sprague-Dawley rats reported prominent clusterin-ir in the contused cortex from 4 d to 2 wk post-injury, whereas no or very low levels of clusterin-ir was observed in the uninjured contralateral cortex and in control animals^16^. In another study, Iwata *et al*. demonstrated prominent clusterin-ir in the ipsilateral cortex from 2 d to 2 wk, and in the ipsilateral thalamus from 1 month to 6 months post-injury in a parasagittal FPI model in adult male Sprague-Dawley rats^32^. In the CCI model in adult male C57BL/6J mice^34^, Western blot analysis showed clusterin up-regulation in the injured brain tissue already at 6 h post-injury, which was sustained for 5 days. Troakes *et al*. investigated several cortical and subcortical areas in humans who died from closed head injury and found increased clusterin expression in the white matter^33^. Like in the present experimental study, abnormal clusterin expression was also long-lasting in humans for up to 10 months post-injury. In the present study, we also observed clusterin expression in the hippocampus, which has not been reported previously. These data indicate that clusterin expression is induced by different types of impact forces. Moreover, the TBI-induced clusterin expression in humans can be recapitulated in rodent models of TBI.

Our study shows that TBI-induced clusterin expression is not located in cellular compartments and is extracellular. Data from our colocalisation study show that punctate clusterin-ir was not located in the neuronal somata, astrocytes, or microglial cells. This contradicts findings in the parasagittal FPI model^32^, where clusterin colocalised with NeuN and GFAP. Also, in the standardised weight-drop model, clusterin-ir overlapped with MAP5-labelled neuronal perikarya and GFAP-positive astrocytes^16^. Similar to the human study by Troakes *et al*.^33^, we observed clusterin-ir adjacent to, but not colocalised with, microglial markers CD68 and OX-42. Troakes *et al*. also observed clusterin-ir astrocytes, particularly in cases with long post-TBI survival. As oxidative stress is a hallmark pathology after TBI, and some studies have demonstrated the role of clusterin in altering the mitochondrial apoptosis pathways via its interaction with BAX, Ku70, and Bcl-xl^19–21,38^, we also assessed the colocalisation of punctate clusterin-ir with the mitochondrial marker MT-CO1, but observed no mitochondrial colocalisation. These studies reveal differences in cellular vs extracellular localisation of post-injury clusterin in different models. Whether these differences relate to the timing of sampling, model/preparation analysed, or antibody used for immunohistochemistry remains to be investigated.

Although we did not observe clusterin-ir in white matter tracts such as the corpus callosum or internal capsule as reported by Troakes *et al*.^33^, we observed robust labelling in layer IV of the perilesional cortex at 7 d post-injury. Previous studies demonstrated that the ventroposterior nucleus of the thalamus sends a heavy projection to layer IV of the cerebral cortex^39^, where we observed the intense punctate clusterin-ir. We also observed prominent clusterin-ir in the vicinity of the activated microglia in the thalamus, indicating a potential role of clusterin in pinpointing the chronic post-injury neuroinflammation in the thalamo-cortical axis. As the clusterin mRNA levels were increased in both the perilesional cortex and thalamus, these data suggest that the clusterin-ir was due to ongoing synthesis rather than its accumulation over time.

We also observed prominent punctate clusterin-ir in the stratum lacunosum of CA1 and molecular layer of the dentate gyrus, which are the terminal fields of the medial perforant pathway^40^. Interestingly, there was no immunopositivity in the entorhinal cortex or angular bundle. Unlike in the perilesional cortex and thalamus, clusterin mRNA expression was not robustly increased in the hippocampus. It remains to be further explored whether the mechanism of clusterin accumulation related to thalamo-cortical pathology differs from that in the dentate gyrus.

Finally, we observed clusterin-ir in the hypothalamus, a finding that is also reported in other rodent studies^41,42^. In contrast to the injury-area specific expression in the cortex, hippocampus, or thalamus, hypothalamic clusterin expression appeared constitutive as it was observed bilaterally in the neuronal somata in the paraventricular and supraoptic nuclei of the hypothalamus in both control and injured animals, as well as in the dendrites and axons.

Taken together, in injured areas of the cortex, hippocampus, and thalamus, punctate clusterin-ir appeared extracellular. In normal and injured hypothalamus, however, the staining was exclusively intraneuronal.

Our immunohistochemical and RT-qPCR analysis revealed a robust and long lasting increase in brain clusterin mRNA and protein expression in response to TBI. Contrary to our expectations, our analysis of circulating plasma clusterin levels from blood sampled via cardiac puncture showed a 15% reduction in plasma clusterin levels at 2–6 h post-TBI compared with controls. To the best of our knowledge, our study is the first to investigate the temporal evolution of post-TBI clusterin expression in the brain and its corresponding levels in the plasma and CSF. There could be several reasons for uncoupling of post-TBI brain and plasma clusterin expression. The brain expression of clusterin in the TBI model was predominantly extracellular, with no prominent vascular immunoreactivity. Since molecules expressed in the damaged neurovascular unit could be expected to reach the circulation more likely^7,43^, the extracellular non-vascular location of clusterin in injured brain could be one of the factors explaining its low levels in circulation. The extracellular clusterin is also susceptible to rapid degradation by the proteinases in the extracellular space^44^, prior to its release in circulation. Further, the time window of the expression of a molecular biomarker in peripheral circulation might not match with its relative peak expression in the brain tissue, particularly if the release is immediate after the exposure of the brain to the impact force. Clusterin is constitutively expressed in almost all mammalian tissues, with abundant presence in biofluids^15^. Thus, the TBI-related release from the brain can become diluted in the peripheral circulation, and this, more challenging to detect^45^. It also remains to be investigated if tail-vein plasma would reflect a different expression pattern of clusterin as compared to the cardiac plasma used in this study. Finally, the plasma clusterin levels were analyzed in this study with a traditional ELISA. The recently developed more sensitive technologies like single molecule enzyme-linked immunosorbent assay (SiMoA), which is ~1000-fold more sensitive than the traditional ELISA^46^, could be more suitable for detection of small pathology-specific alterations of constitutively expressed proteins in the circulation. However, SiMoA currently works best with predesigned antibody panels, which do not include clusterin assay.

In contrast to plasma, our iTRAQ proteomics analysis found a 1.3-fold elevated CSF clusterin level at 12 months after lateral FPI along with a chronically elevated clusterin expression in the injured brain areas of the same animals. This indicates that the CSF might be a better source to reflect the TBI induced altered clusterin protein expression in the brain than peripheral circulation. However, since sampling the CSF is an invasive procedure for both animals and humans, it has a limited applicability in identification of non-invasive biomarkers.

The uncoupled pattern of clusterin expression in tissue and circulation has also been observed in other neuropathologies. For instance, previous studies of patients with epilepsy demonstrated increased clusterin mRNA expression levels in the temporal lobe tissue^47^. Still, several epilepsy studies have shown a 20–30% reduction in serum clusterin levels in patient with “idiopathic temporal lobe epilepsy” or “idiopathic epilepsy”^27,28^. So far, none of the previous studies has analysed clusterin as a biomarker for post-traumatic epilepsy. Our preliminary analysis, which was powered to detect significance if the AUC was 0.900 or higher did not support the idea that plasma clusterin would differentiate rats with or without post-traumatic epilepsy. In AD, several studies reported increased clusterin-ir in amyloid plaques^48–50^. Some studies analysing serum/plasma levels of clusterin in AD reported a 1.1-fold increase in plasma clusterin levels, whereas others reported a 1.4–1.7 fold reduction or no significant difference^24,25,29–31,51–53^. These studies suggested that increased levels of clusterin could serve as biomarkers for more severe cognitive decline, increased risk of AD, rate of AD progression, entorhinal cortex atrophy, baseline disease severity, as well as behavioural characteristics such as agitation, aggression, and decreased risk of dementia and stroke in younger non-demented participants. An iTRAQ proteomic analysis of plasma in APOE transgenic mice with mild but not severe CCI–induced TBI revealed a 1.4-fold elevation in clusterin levels at 1 month post-injury^35^. No corresponding brain expression pattern of clusterin was reported. In acute ischemic stroke^26^, clusterin overexpression was observed in the cortex, hippocampus, and striatum of ischemic C57BL/6J mice, but no serum/plasma analysis was performed in the mouse model. The same study analysed serum clusterin levels in human ischemic stroke patients and observed a 1.16-fold elevation. Taken together, the above findings indicate that alterations in circulatory clusterin levels are observed in multiple neuropathologies and in both directions.

In humans, CSF analysis of clusterin typically revealed a 20–80% reduction in drug-responsive and drug-refractory epilepsy patients with temporal lobe epilepsy compared with controls^27,28^. On the other hand, patients with relapsing–remitting multiple sclerosis with clinically-isolated syndrome reported a 30–67% increase in CSF clusterin^54^. Jongbloed *et al*. simultaneously analysed plasma and CSF clusterin levels in AD, and identified a poor correlation between the two^55^, and poor performance of CSF clusterin as a diagnostic or prognostic AD marker^24^.

Thus, reports on AD typically show increased levels of clusterin around amyloid plaques in the brain, plasma/serum, and CSF, although no studies have investigated the different tissues in the same subject, and contradictory findings exist. In patients and in animal model studies of epilepsy and TBI, as well as in the present experimental TBI study, the match in clusterin levels between different tissues is less apparent. Whether this relates to differential regulation of clusterin expression over time in different disease pathologies or different transport mechanisms of clusterin from the brain parenchyma to blood requires further investigation to optimise the use of circulating clusterin as a biomarker for disease outcome.

## Conclusion

Our findings indicate consistent, region-specific, and prolonged overexpression of clusterin protein extracellularly in the injured brain areas, consistent with increased clusterin mRNA levels and clusterin protein levels in the CSF. In contrast to our expectations, analysis of cardiac plasma clusterin levels revealed an acute post-injury reduction rather than an increase.

## Materials and Methods

**Figure 5:**
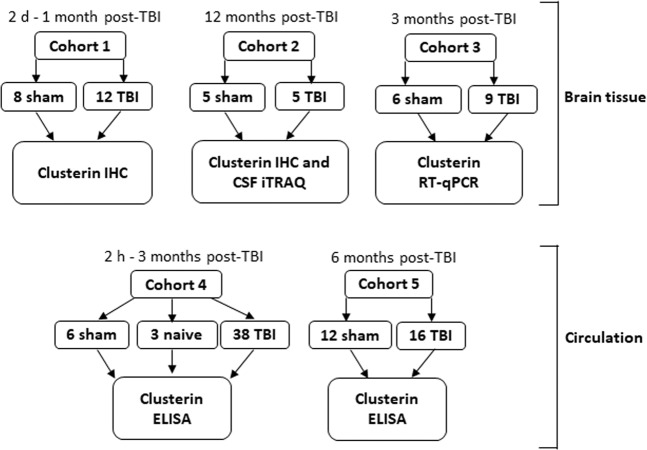
Study design for clusterin analysis in brain tissue, plasma, and CSF. Spatiotemporal expression of clusterin protein in the brain after TBI was investigated in cohorts 1–2. Chronic expression of clusterin mRNA in the brain was assessed using RT-qPCR in cohort 3. Post-TBI plasma clusterin levels were assessed using ELISA in cohorts 4–5. In cohort 5, 5 of the 16 TBI rats had epilepsy. Consequently, the study was powered to diagnose post-traumatic epilepsy in the TBI group if the AUC was ≥0.900. Finally, clusterin levels in CSF was analysed using iTRAQ proteomics in the chronic cohort 2. Abbreviations: CSF, cerebrospinal fluid; d, days; ELISA, enzyme-linked immunosorbent assay; h, hours; IHC, immunohistochemistry; iTRAQ, isobaric tag for relative and absolute quantification; RNA-Seq, RNA sequencing; RT-qPCR, reverse transcription-quantitative polymerase chain reaction; sham, sham-operated controls; TBI, traumatic brain injury.

The study design and description of the five animal cohorts included in the study are shown in Fig. 5.

### Animals

Altogether, 120 adult male Sprague-Dawley rats (297–454 g, Harlan Laboratories S.r.l., The Netherlands or Italy) were used (80 TBI, 37 sham-operated experimental controls, 3 naïve). Animals were housed in a controlled environment (temperature 22 ± 1 °C; humidity 50–60%; 12-h light/dark cycle). Water and pellet food were provided *ad libitum*. Animal procedures were approved by the Animal Ethics Committee of the Provincial Government of Southern Finland and carried out in accordance with the guidelines of the European Community Council Directives 2010/63/EU.

**Cohort 1** (12 TBI, 8 sham) was used to assess the spatio-temporal evolution of clusterin immunoreactivity (ir) in the brain tissue during acute and subacute time-points after TBI. Rats were perfused for histology at 2 d, 1 wk, 2 wk and 1 month post-TBI (3 TBI and 2 sham each time-point). **Cohort 2** was followed up for up to 12 months to assess the chronic expression of clusterin in the brain (5 TBI, 5 sham). At the end of the follow-up, the rats were cannulated to measure CSF clusterin levels in the cisterna magna. To investigate whether elevated clusterin protein levels in the brain tissue were associated with elevated *Clu* gene expression, RT-qPCR analysis for *Clu* mRNA was performed in **cohort 3** (9 TBI, 6 sham). The rats were killed at 3 months post-TBI and the perilesional cortex and ipsilateral thalamus was sampled. In **cohort 4** (38 TBI, 6 sham and 3 naïve), plasma clusterin levels were assessed at 2 h, 6 h, 1 d, 2 d, 3 d, 5 d, 1 wk, 2 wk, 1 month, and 3 months post-TBI (3 TBI animals per time-point for 2 h-2 d, 4 TBI animals per time-point for 3 d-1 month, and 6 TBI rats for 3 months). **In cohort 5**, we analysed the plasma clusterin levels at 6 months post-TBI (16 TBI, 12 sham).

### Induction of TBI with lateral fluid-percussion

Lateral FPI-induced TBI was performed as described previously^56,57^. During the procedure, animals were anaesthetised with an intraperitoneal injection (6 ml/kg) of a mixture containing sodium pentobarbital (58 mg/kg), magnesium sulfate (127.2 mg/kg), propylene glycol (42.8%), and absolute ethanol (11.6%). The anaesthetic cocktail in cohorts 1, 2, 3, and 4 also contained 60 mg/kg chloral hydrate, but chloral hydrate was omitted from the cocktail used for the more recent cohort 5 as its use is no longer permitted. The impact pressure (atm) and the weight of the animals included in the study at the time of TBI induction (g) were as follows: **cohort 1:** 3.17 ± 0.03 atm (303–329 g), **cohort 2:** 3.18 ± 0.01 atm (323–381 g), **cohort 3:** 3.32 ± 0.01 atm (336–371 g), **cohort 4:** 3.34** ± **0.01 atm (327–454 g), **cohort 5:** 3.25** ± **0.02 atm (331–419 g). Sham-operated experimental controls received anaesthesia and underwent all surgical procedures without lateral FPI. Naïve animals did not undergo any surgical procedures or anaesthesia.

#### Electrode implantation and detection of pentylenetetrazol (PTZ)-induced seizure susceptibility in cohort 5

To monitor the occurrence of spontaneous seizures after lateral FPI, the rats from cohort 5 were implanted with three skull electrodes, one reference and one ground electrode at 5 months post-TBI as described previously^58^. Continuous (24/7) 1 month video-electroencephalography (EEG) monitoring was performed. Seizure susceptibility was tested with a PTZ test at 6 months post-TBI, prior to killing the rats^59^. After a baseline video-EEG recording, the animals were injected with PTZ (25 mg/kg, i.p., Sigma-Aldrich YA-Kemia Oy, Finland) dissolved in 0.9% saline (final concentration 18.0 mg/ml) and continuously video-EEG monitored for 60 min. The video-EEG files were then analysed visually and with an automated seizure detection algorithm^58^.

### Clusterin immunohistochemistry

#### Fixation and tissue processing

For immunohistochemistry, rats were anaesthetized with the same anaesthesia cocktail used during TBI induction (cohorts 1 and 2). The animals were transcardially perfused with 0.9% saline [30 ml/min at room temperature (RT)] for 3 min followed by 4% paraformaldehyde in 0.1 M sodium phosphate buffer, pH 7.4 (30 ml/min at 4 °C), for 30 min. The brain was removed from the skull and post-fixed in 4% paraformaldehyde for 4 h (at 4 °C), and then cryoprotected in a solution containing 20% glycerol in 0.02 M potassium phosphate buffered saline for 24 h. The brains were frozen on dry ice and stored at −70 °C until cut. The brains were sectioned in a coronal plane (30-µm-thick sections; 1-in-10 series for cohort 1 and 1-in-8 series for cohort 2) with a sliding microtome (Leica SM 2000, Leica Microsystems Nussloch GmbH, Nussloch, Germany). The first series was collected in 10% formalin for thionin staining and stored at RT. The remaining series were stored in tissue-collecting solution (TCS: 30% ethylene glycol, 25% glycerol in 0.05 M sodium phosphate buffer) at −20 °C until staining.

#### Single-immunolabelling

Immunohistochemistry was performed as previously described^60^. In cohort 1, primary antibody incubation was performed with a rabbit polyclonal antibody raised against clusterin (1:1000, #STJ92346, St John’s Laboratory, London, UK). In cohort 2, primary antibody incubation was performed with a mouse monoclonal antibody raised against clusterin (1:4000, #LS-B3762, LifeSpan Biosciences, Inc., Seattle, WA, USA, for cohort 2). All primary antibody incubations were performed in potassium phosphate buffered saline at 4 °C for 2–3 nights. The two clusterin primary antibodies used in cohorts 1 and 2 produced comparable immunolabelling.

#### Double-labelling immunofluorescence

To identify the cellular and/or intracellular localization of the clusterin immunostaining, double-labelling was performed with the neuronal marker NeuN, astrocyte marker GFAP, microglial markers CD68 and OX-42, and the mitochondrial marker MT-CO1. The rabbit polyclonal clusterin primary antibody (#STJ92346) was used at a concentration of 1:800 for all immunofluorescence labelling. Primary antibody concentrations for the cellular markers were as follows: anti-NeuN (MAB 377, Millipore, 1:2000), anti-GFAP (GmBH, 1:2000), anti-CD68 (MAB 1435, Millipore, 1:1000), anti OX-42 (Serotec MCA275G, 1:2000), and anti MT-CO1 (ab14705, Abcam, 1:500).

### *Clu* mRNA RT-qPCR

#### Sampling of brain tissue for *Clu* mRNA expression analysis

The protocols for brain tissue sampling at 3 months post-TBI (cohort 3) and RNA extraction from the perilesional cortex and ipsilateral thalamus has been described previously^36^.

#### RT-qPCR analysis of *Clu* mRNA expression

Expression pattern of *Clu* mRNA in the perilesional cortex and ipsilateral thalamus was analysed with TaqMan RT-qPCR. RNA samples were adjusted to a final concentration of 10 ng/µl, and cDNA synthesis was performed with a high-capacity RNA-to-cDNA kit (#4387406, ThermoFisher Scientific). Samples were stored at −20 °C until further processed. Prior to RT-qPCR reaction, cDNA samples were diluted to 2 ng/µl concentration, and 12 ng was added to the final reaction volume of 20 µl. RT-qPCR was run using StepOnePlus™ Real-Time PCR System (Software v2.2.2, Applied Biosystems). C_t_ values were normalized to the housekeeping gene GAPDH (Rn99999916_s1, ThermoFisher Scientific) with the formula 2^−ΔCt^.

### Enzyme-linked immunosorbent assay (ELISA) of plasma clusterin levels

#### Cardiac puncture

In cohorts 4 and 5, cardiac plasma was collected when animals were killed at 2 h, 6 h, 1 d, 2 d, 3 d, 5 d, 1 wk, 2 wk, 1 month, 3 months or 6 months post-TBI, preceding the perfusion-fixation. Of note, for cohort 5, PTZ injection for the seizure susceptibility test was performed 2 h before killing and cardiac blood sampling. The rats were anaesthetized with the same anaesthesia cocktail used during TBI induction, and cardiac blood was withdrawn. The blood samples were centrifuged at 3400 rpm (RT) or 1300 g (4 °C) for 10 min. Plasma was pipetted into 50-µl aliquots (0.5 ml Protein LoBind tubes, ref: 0030108094, Eppendorf) and stored at −70 °C.

#### Quantification of haemolysis

We quantified the plasma haemolysis by measuring the absorbance of haemoglobin at 414 nm using a NanoDrop^TM^ 1000 spectrophotometer.

#### Clusterin ELISA

First, we prepared a 4-point plasma dilution curve (1:100, 1:500, 1:1000, 1.2000) to assess the linearity within the sensitivity range of the assay. Consequently, one plasma aliquot tube per animal was melted, analysed for haemolysis, and diluted 1:500 in PBS to be used in ELISA. Duplicate samples from each rat and standards were analysed with the rat clusterin ELISA kit (#ERCLU, ThermoFisher Scientific), according to manufacturer’s instructions. To control the batch (plate-to-plate variation) effect, plasma samples from the same two sham-operated animals of cohort 4 were included in each ELISA run. Consequently, clusterin concentrations from all samples in each ELISA plate was normalised to the average concentration of the two controls from the same plate.

### CSF proteomics

#### Cannulation of cisterna magna

CSF proteomic analysis was performed at 12 months post-TBI in cohort 2. Cannula for CSF sampling was implanted at 11.5 months post-TBI, and sampling began 1 wk after the implantation. Only the CSF samples without apparent blood contamination by visual inspection were selected for analysis.

#### Proteomics

Quantitative proteomics was performed with the isobaric tags for relative and absolute quantitation (iTRAQ) method. For iTRAQ, 20 μg of CSF protein from the 5 sham rats and the 5 TBI rats were pooled to obtain 100 μg of CSF protein for both sham and TBI groups. Details of the iTRAQ procedure are described in the Supplementary Material.

### Statistical analysis

Statistical analyses were performed using IBM SPSS Statistics 25.0 (IBM Corp., Armonk, NY, USA). Graphs were prepared with GraphPad Prism (version 8.0.1, GraphPad software) Comparisons of three or more groups were done using the non-parametric Kruskal-Wallis ANOVA test followed by *post hoc* analysis with the Mann-Whitney *U* test. For linear regression analysis, normal distribution of the data was analysed with the Shapiro-Wilk test. If the data were not normally distributed, they were log-transformed to obtain a normal distribution, followed by the regression analysis. A receiver operating characteristic (ROC) test was performed to investigate the sensitivity and specificity of plasma clusterin level as a biomarker for TBI. A P-value less than 0.05 was considered statistically significant.

## Supplementary information

Supplementary information.

## Data Availability

The datasets generated and/or analysed during the current study are available from the corresponding author on reasonable request.
