# Supplementary material for: Dynamics of clusterin protein expression in the brain and plasma following experimental traumatic brain injury
Source: Sci Rep. 2019 Dec 27;9:20208. doi: 10.1038/s41598-019-56683-6 (PMC6934775; doi:10.1038/s41598-019-56683-6)
Supplement: Supplementary file 1 — Supplementary information. [file 41598_2019_56683_MOESM1_ESM.pdf]

Dynamics of clusterin protein expression in the brain and plasma following experimental traumatic brain injury

Shalini Das Gupta[1], Anssi Lipponen[1], Kaisa M.A. Paldanius[1], Noora Puhakka[1], Asla Pitkänen[1*]

[1]*A. I. Virtanen Institute for Molecular Sciences, University of Eastern Finland, PO Box 1627, FI-70211 Kuopio, Finland*

***Corresponding author:** Asla Pitkänen, MD, PhD, A. I. Virtanen Institute for Molecular Sciences, University of Eastern Finland, PO Box 1627, FI-70211 Kuopio, Finland, Tel: +358-50-517 2091, Fax: +358-17-16 3030, E-mail: asla.pitkanen@uef.fi

Supplementary methods

Assessment of long-term changes in CSF proteomics after TBI

Cannula implantation into the cisterna magna for CSF sampling. At 11.5 months post-TBI, the rats in cohort 1 were anaesthetized and mounted in a stereotactic frame. The skull was exposed and cleaned with sterile 0.9% NaCl. For supporting screws, two holes were drilled into the skull. Then, a 2-mm-diameter hole was drilled at the midline caudal to the interparietal suture for the cannula. The cannula comprised two polyethylene tubes glued together (outer tube Ø2 mm and inner tube Ø 1 mm; Intramedic, Becton Dickinson)[1]. The cannula was inserted into the cisterna magna, fixed with dental acrylic, and closed with a plug made from an injection needle.

Collection of CSF from the cisterna magna. CSF was collected beginning 1 wk after cannula implantation at 12 months post-TBI. Samples of cisternal CSF (50-150 μl) were slowly withdrawn into the syringe connected to a tube attached to the cannula. During sample collection, the rat was allowed to move freely. Each animal was sampled 1-4 times, and the delay between the sampling was 1 to 3 d. Samples were collected into a sterile Eppendorf® tube and immediately centrifuged (2000 x *g*) for 2 min in a micro centrifuge (Galaxy Mini VWR International) to harvest the blood cells. The supernatant was transferred into a clean Eppendorf® tube and placed on ice. The CSF was then centrifuged 1500 x *g* for 10 min at +4^{0}C, transferred into a clean Eppendorf® tube, and frozen at -70^{0}C until assayed. Only transparent CSF samples without apparent blood contamination by visual inspection were used for the analysis.

Isobaric Tag for Relative and Absolute Quantitation (iTRAQ). For iTRAQ analysis, 20 μg of CSF protein from 5 control and 5 TBI rats were pooled, resulting in 100 μg of CSF protein from the controls and 100 μg from the TBI animals. Albumin and IgG were removed

using an albumin and IgG removal kit (#RPN6300, GE Healthcare) according to the manufacturer's protocol. After acetone precipitation, air-dried protein pellets were resuspended in 20 µl of 500 mM triethyl ammonium bicarbonate buffer, pH 8.5. Each of the CSF protein samples was dissolved in the triethyl ammonium bicarbonate buffer provided with the 4-plex iTRAQ kit (Applied Biosystems). Samples were then treated with a reducing reagent, and cysteine residues were blocked according to the manufacturer's instructions (Applied Biosystems). CSF protein samples were digested with 5 µl of 1 µg/µl freshly prepared sequence-grade-modified trypsin (Promega) at 37^{0}C overnight and labelled with the iTRAQ tag. Protein samples were labelled as follows: Sham-operated control iTRAQ 114 and animals with TBI iTRAQ 116. Next, prior to pI-based peptide pre-fractionation, the iTRAQ–labelled peptide samples were pooled together and desalted using Empore C18 disks. Desalted and dried iTRAQ peptides were resuspended in 125 µl of isoelectric focusing buffer (4 M urea, 2% IPG buffer pH 3-10) and fractionated on a 7-cm IPG strip pH 3-10 using IPGphor program: Step 1: rehydration 12 h at 20^{0}C; Step 2: hold 500 V for 1 h; Step 3: gradient 500 V – 1000 V 15 min; Step 4: hold 1000 V 1 h; Step 5: gradient 1000 V – 8000 V 30 min; Step 6: 8000 V 2 h; max 50 µA per strip.

After isoelectric focusing, the IPGphor strip was washed in petroleum ether for 2 × 10 s to remove excess oil. Thereafter, iTRAQ-labelled peptides were extracted from the strip. Briefly, the IPGphor strip was cut into three pieces, and peptides were extracted according to Cargile et al.[2]. Each piece was immersed in 0.1% trifluoroacetic acid (TFA) in H_2O for 15 min at 37°C. The solution was transferred into a clean plastic Eppendorf tube and the strip was soaked in 0.1% TFA in 50% acetonitrile for 15 min at 37^{0}C. The solution was collected, and the strip incubated in 0.1% TFA in 100% acetonitrile for 15 min at 37^{0}C. Solutions from each piece of the IPGphor strip were combined and evaporated to dryness using SpeedVac (RC1010 Jovan, Berner). Analysis of iTRAQ–labelled peptides was performed in the

University of Turku Proteomic Core Facility. Briefly, liquid chromatography–tandem mass spectroscopy analyses were performed using an integrated LC system (Famos, SwitchosII and Ultimate, LC Packings/MDs Sciex, Sunnyvale, CA) coupled to a QSTAR Pulsar *i* mass spectrometry (Applied Biosystems/MDS Sciex, Toronto, Canada). Peptides were first concentrated on a pre-column (0.3 x 5 mm PepMap C18, LC Packings) and chromatographic separation was achieved on a 15-cm C18 column (75 µm x 15 cm, Magic 5 µm 100 Å C_{18}, Michrom BioResources Inc.). A linear 90-min gradient (from 2% to 34% acetonitrile) was used to elute peptides. The flow rate was 200 nl/min. The information-dependent acquisition method consisted of a TOFMS survey scan of mass range 350-1500 amu and two product ion scans of mass range 50-2000 amu. The two most intense peaks over 10 counts with a charge state of 2-3 were selected for fragmentation. Protein Pilot software (version 2.0.1, Applied Biosystems) was used for protein identification and quantification. Database searches were performed against Swiss Prot – Trembl (version SProt 54.6 and Tembl 37.6), taxonomy rat.

Supplementary figures and tables

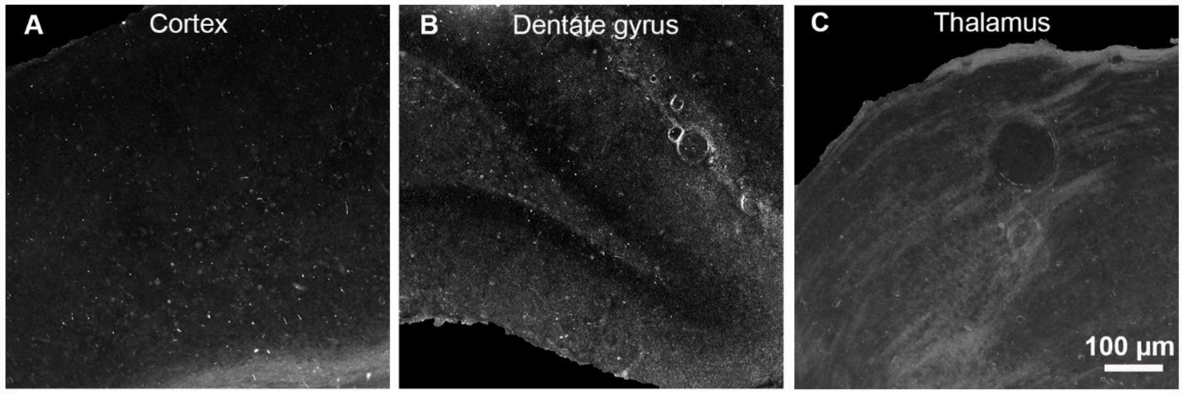

Supplementary Figure 1. Representative darkfield photomicrographs of clusterin-immunostained coronal sections of the brain. Unlike in injured brains, no punctate clusterin-immunoreactivity was found in the **(A)** ipsilateral cortex, **(B)** ipsilateral dentate gyrus and **(C)** ipsilateral thalamus of a sham-operated rat. Scale bar equals 100 µm (all panels).

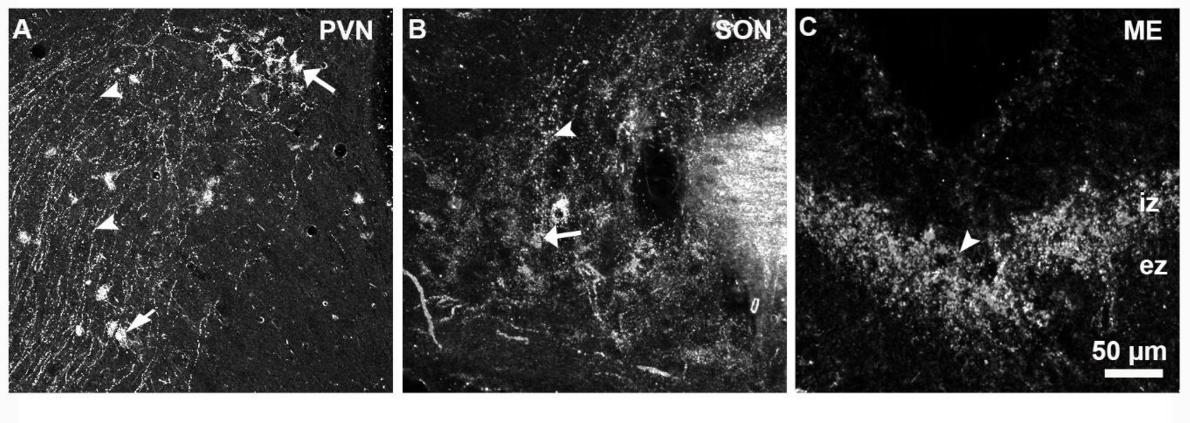

Supplementary Figure 2. Constitutive expression of clusterin bilaterally in the hypothalamus in sham-operated and TBI cases. A representative coronal darkfield photomicrograph of clusterin-ir in the **(A)** paraventricular nucleus (PVN, arrow) and also in neurons scattered in the more lateral hypothalamus (small arrow), **(B)** supraoptic (SON, arrow) nucleus and **(C)** in the median eminence (ME, arrowhead) of the hypothalamus in a sham-operated rat. Immunoreactivity was observed in both the neuronal somata (*e.g.,* arrows in A and B) and varicose axons (arrowheads in A-C). Comparable clusterin-ir was observed on the contralateral side, and also in TBI rats from 2d – 1 month post-injury. Abbreviations: ez, external zone of median eminence; iz, internal zone of median eminence, ME, median eminence; PVN, paraventricular nucleus; SON, supraoptic nucleus. Scale bar = 50 µm for all panels.

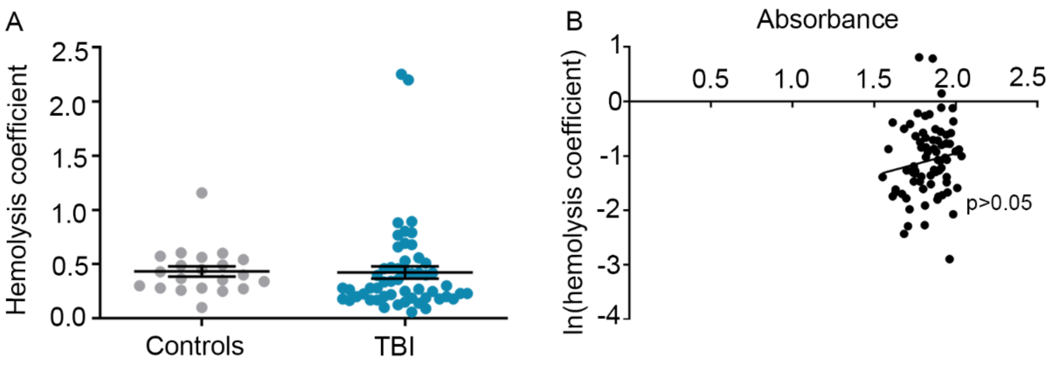

Supplementary Figure 3. Plasma clusterin level in cardiac plasma is unaffected by haemolysis. (A) Haemolysis coefficients (measured at 414 nm) did not differ between controls and TBI animals ($p>0.05$). **(B)** Moreover, linear regression analysis revealed no significant association between the plasma haemolysis coefficient and clusterin ELISA absorbance ($p>0.05$).

Supplementary Table 1. iTRAQ revealed 12 proteins in the cisternal cerebrospinal fluid (CSF) that were differentially expressed (nominal p<0.05) between the control and TBI rats at 12 months post-injury.

NCBI Accession	Name	TBI/Control	Nominal p-value	FDR-corrected p-value
**P01026|CO3_RAT**	**Complement C3 precursor**	**1.08**	**0.02**	**0.11**
Q63041|A1M_RAT	Alpha-1-macroglobulin precursor	1.02	0.52	0.70
P14046|A1I3_RAT	Alpha-1-inhibitor 3 precursor	1.01	0.54	0.70
**Q7TMC7|Q7TMC7_RAT**	**Ab2-417**	**0.83**	**6.59E-07**	**1.68E-05**
P05545|SPA3K_RAT	Serine protease inhibitor A3K precursor	1.04	0.55	0.70
P09006|SPA3N_RAT	Serine protease inhibitor A3N precursor	0.96	0.47	0.68
P17475|A1AT_RAT	Alpha-1-antiproteinase precursor	1.01	0.72	0.82
P20059|HEMO_RAT	Hemopexin precursor	0.94	0.31	0.53
**P02091|HBB1_RAT**	**Hemoglobin subunit beta-1**	**1.38**	**0.003**	**0.03**
Q6MG79|Q6MG79_RAT	Complement component 4, gene 1	1.13	0.14	0.32
**P01946|HBA_RAT**	**Hemoglobin subunit alpha-1/2**	**1.40**	**1.67E-09**	**8.52E-08**
P06238|A2MG_RAT	Alpha-2-macroglobulin precursor	1.01	0.91	0.93
**P08932|KNT2_RAT**	**T-kininogen 2 precursor**	**0.64**	**0.01**	**0.07**
P01015|ANGT_RAT	Angiotensinogen precursor	1.26	0.12	0.32
Q01177|PLMN_RAT	Plasminogen precursor	0.92	0.24	0.44
P14841|CYTC_RAT	Cystatin-C precursor	0.94	0.38	0.57
P04937|FINC_RAT	Fibronectin precursor	1.12	0.08	0.27
**Q6PAH0|Q6PAH0_RAT**	**Apolipoprotein E**	**1.28**	**0.04**	**0.17**
Q68FP1|GELS_RAT	Gelsolin precursor	1.05	0.60	0.75
Q68FY4|Q68FY4_RAT	Group specific component	0.92	0.17	0.33
P06866|HPT_RAT	Haptoglobin precursor	1.07	0.35	0.56
Q9JL97|Q9JL97_RAT	GPI-anchored ceruloplasmin	0.86	0.14	0.32
Q7TMB9|Q7TMB9_RAT	Liver regeneration protein lrryan	0.92	0.38	0.57
Q5EBC0|Q5EBC0_RAT	Inter alpha-trypsin inhibitor, heavy chain 4	1.48	0.16	0.33
Q5M7T5|Q5M7T5_RAT	Serine	0.80	0.14	0.32
**P22057|PTGDS_RAT**	**Prostaglandin-H2 D-isomerase precursor**	**0.84**	**0.0003**	**0.005**
**Q6P7S6|Q6P7S6_RAT**	**Clusterin**	**1.29**	**0.002**	**0.02**
Q64610|ENPP2_RAT	Ectonucleotide pyrophosphatase/phosphodiesterase family member 2 precursor	1.01	0.88	0.93
P02764|A1AG_RAT	Alpha-1-acid glycoprotein precursor	0.70	0.08	0.27
Q6P734|IC1_RAT	Plasma protease C1 inhibitor precursor -	1.34	0.10	0.28
**Q5PQU1|Q5PQU1_RAT**	**Kininogen 1**	**0.78**	**0.007**	**0.05**
Q7TP05|Q7TP05_RAT	Da1-24	1.04	0.66	0.81
**P02651|APOA4_RAT**	**Apolipoprotein A-IV precursor**	**1.07**	**0.02**	**0.11**
Q7TMA9|Q7TMA9_RAT	Ac1262	0.96	0.55	0.70
Q6P7A8|Q6P7A8_RAT	SPARC-like 1	0.89	0.16	0.33
**P04639|APOA1_RAT**	**Apolipoprotein A-I precursor**	**0.76**	**0.04**	**0.17**
Q9QX79|FETUB_RAT	Fetuin-B precursor	0.76	0.15	0.33

Q9ESB2|Q9ESB2_RAT	Histidine-rich glycoprotein	1.01	0.89	0.93
**P36953|AFAM_RAT**	**Afamin precursor**	**0.89**	**0.03**	**0.15**
P07151|B2MG_RAT	Beta-2-microglobulin precursor	0.99	0.88	0.93
Q9QXU9|PCSK1_RAT	ProSAAS precursor	0.97	0.70	0.81
P68136|ACTS_RAT	Actin, alpha skeletal muscle	1.33	0.05	0.20
Q9R1B7|Q9R1B7_RAT	Chromogranin A precursor	1.15	0.29	0.51
Q68FT8|Q68FT8_RAT	Serine	1.04	0.68	0.81
P02767|TTHY_RAT	Transthyretin precursor	1.23	0.09	0.27
Q7TP75|Q7TP75_RAT	Aa2-066	0.96	0.88	0.93
P11517|HBB2_RAT	Hemoglobin subunit beta-2	1.56		
Q6TXE2|Q6TXE2_RAT	LRRGT00057	0.94		
Q63416|ITIH3_RAT	Inter-alpha-trypsin inhibitor heavy chain H3 precursor	0.37		
P13596|NCAM1_RAT	Neural cell adhesion molecule 1, 140 kDa isoform precursor	0.72		
Q63429|Q63429_RAT	Polyubiquitin	1.00	1.00	1.00
Q8R417|Q8R417_RAT	Neuron-specific epidermal growth factor-like repeat domain-containing protein	0.94	0.54	0.70
Q08420|SODE_RAT	Extracellular superoxide dismutase [Cu-Zn] precursor	1.13	0.09	0.27
P00762|TRY1_RAT	Anionic trypsin-1 precursor	0.87	0.32	0.53
Q6PEC5|Q6PEC5_RAT	Putative uncharacterized protein	0.86	0.18	0.34
Q6QI47|Q6QI47_RAT	LRRGT00161	0.10		
Q6P7A4|Q6P7A4_RAT	Prosaposin	1.11		

Proteins identified as differentially expressed are indicated in blue (FDR < 0.05).

Abbreviations: CSF, cerebrospinal fluid; FDR, false discovery rate; iTRAQ, Isobaric Tag for Relative and Absolute Quantitation; TBI, traumatic brain injury.

References

1. Pitkänen, A., Jolkkonen, J. & Riekkinen, P. J. Somatostatin-like immunoreactivity (SLI) in cisternal cerebrospinal fluid of rats kindled by pentylenetetrazol. *Brain Res.* **416**, 180–182 (1987).

2. Cargile, B. J., Bundy, J. L., Freeman, T. W. & Stephenson, J. L. Gel based isoelectric focusing of peptides and the utility of isoelectric point in protein identification. *J. Proteome Res.* **3**, 112–119 (2004).
